# Biodegradation and Prospect of Polysaccharide from Crustaceans

**DOI:** 10.3390/md20050310

**Published:** 2022-05-02

**Authors:** Shuting Qiu, Shipeng Zhou, Yue Tan, Jiayao Feng, Yan Bai, Jincan He, Hua Cao, Qishi Che, Jiao Guo, Zhengquan Su

**Affiliations:** 1 Guangdong Engineering Research Center of Natural Products and New Drugs, Guangdong Provincial University Engineering Technology Research Center of Natural Products and Drugs, Guangdong Pharmaceutical University, Guangzhou 510006, China; q743160056@163.com (S.Q.); zszs64235@163.com (S.Z.); yytan33@163.com (Y.T.); fjy54525452@163.com (J.F.); 2 Guangdong Metabolic Disease Research Center of Integrated Chinese and Western Medicine, Key Laboratory of Glucolipid Metabolic Disorder, Ministry of Education of China, Guangdong TCM Key Laboratory for Metabolic Diseases, Guangdong Pharmaceutical University, Guangzhou 510006, China; 3 School of Public Health, Guangdong Pharmaceutical University, Guangzhou 510310, China; angell_bai@163.com (Y.B.); hejincan300@163.com (J.H.); 4 School of Chemistry and Chemical Engineering, Guangdong Pharmaceutical University, Zhongshan 528458, China; caohua@gdpu.edu.cn; 5 Guangzhou Rainhome Pharm & Tech Co., Ltd., Science City, Guangzhou 510663, China; cheqishi@rhkj.com.cn

**Keywords:** chitinase, chitosanase, chitin deacetylase, chitosan oligomers, enzymatic modification

## Abstract

Marine crustacean waste has not been fully utilized and is a rich source of chitin. Enzymatic degradation has attracted the wide attention of researchers due to its unique biocatalytic ability to protect the environment. Chitosan (CTS) and its derivative chitosan oligosaccharides (COSs) with various biological activities can be obtained by the enzymatic degradation of chitin. Many studies have shown that chitosan and its derivatives, chitosan oligosaccharides (COSs), have beneficial properties, including lipid-lowering, anti-inflammatory and antitumor activities, and have important application value in the medical treatment field, the food industry and agriculture. In this review, we describe the classification, biochemical characteristics and catalytic mechanisms of the major degrading enzymes: chitinases, chitin deacetylases (CDAs) and chitosanases. We also introduced the technology for enzymatic design and modification and proposed the current problems and development trends of enzymatic degradation of chitin polysaccharides. The discussion on the characteristics and catalytic mechanism of chitosan-degrading enzymes will help to develop new types of hydrolases by various biotechnology methods and promote their application in chitosan.

## 1. Introduction

**Figure 1:**
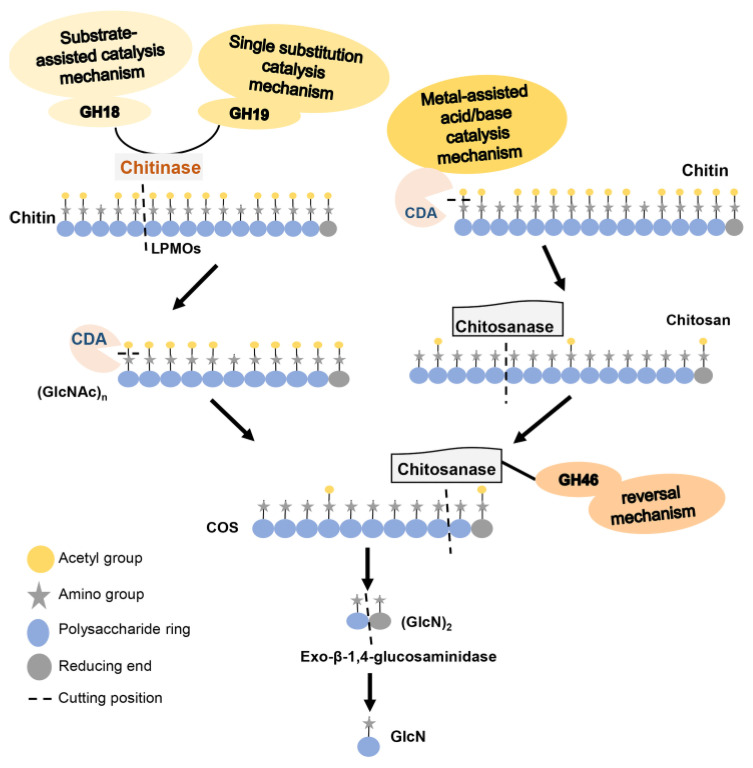
Process of enzymatic degradation of chitosan.

Polysaccharides from renewable biomass are attracting increasing attention. Chitin is the only positively charged natural polysaccharide with a storage capacity second only to cellulose in nature. Although chitin does not accumulate in the marine environment due to the release of energy by microbial degradation, the interspecific substrate cross-feeding cascades also showed that chitin degradation was related to biogeochemical cycling across marine environments. However, there are still a lot of abandoned chitin resources in the ocean [1]. The development and utilization of the resulting chitin resources have extremely high environmental benefits. Chitin, which exists in nature, is a complex of lipids, proteins and bio-minerals, which can be purified by deproteinization and demineralization [2]. Purified chitin can be obtained from the shells of crustaceans, such as shrimp and crabs. There are three crystalline forms: α-chitin, β-chitin and γ-chitin [3]. The difference lies in the arrangement of molecular chains in the crystal cell. α-Chitin is the dominant form, which is formed by a linear arrangement of polysaccharide chains in an antiparallel manner and is highly compressed; the crystallinity is high, and the structure is stable and not easily degradable. β-Chitin is formed by the arrangement of two parallel chains in the same direction [4]. The content of β-chitin is small, and it can be obtained by the extraction method. In contrast, γ-chitin is composed of three chains, two of which are in the same direction and one of which is in the opposite direction, to form the polymerization structure. The polymerization chains are randomly distributed, and the content of γ-chitin is the lowest [5]. The highly extended hydrogen-bond semicrystal structure in chitin makes it difficult to dissolve in common dilute acidic, alkaline and organic solvents. In particular, 50% deacetylated chitin is soluble in water, suggesting that water-soluble chitin (WSC) can be obtained by carefully controlling the deacetylation of chitin [6]. To solve the problem that its molecular weight is too large and insoluble in water, the degree of polymerization of chitin can be reduced by an enzymatic method (Figure 1), and the utilization of chitin can be improved. Finally, glucosamine can be obtained by the degradation of chitosan with Exo-β-1,4-glucosaminidase [7].

Chitosan is a derivative formed by the deacetylation of chitin molecules, which is composed of 2-acetylamino-2-deoxy-β-D-glucopyranose and 2-amino-2-deoxy-β-D-glucopyranose by the β-1,4 glycosidic bond at a randomly distributed composition relatively unstable point [8]. The sequences and percentages of these two molecules determine the biological properties [9]. The biological activity of chitosan molecules is attributed to amino, hydroxy and oxygen bridge functional groups at the C-2, C-3 and C-6 positions. In particular, the ability of its amino group to form chemical bonds with other substances has been widely studied [10]. Therefore, chitosan can undergo hydrolysis, biodegradation and redox reactions. Although the free amino group produced after the conversion of chitin to chitosan by the deacetylation process improves the solubility of chitosan and makes it soluble in dilute acids, the insolubility of chitosan in water and the high viscosity limit the application of chitosan. At present, chitosan has been extensively studied in materials science, such as being used as drug carrier and film material (see Table 1).

COS with variable degrees of polymerization can be obtained by breaking the glycosidic bond of chitosan. The product is usually composed of less than 20 glucosamines linked by β-1,4 glycosidic bonds. There are three main types of functional groups in the structure of COS: C-2, C-3 and C-6 amino groups (acetylamino), primary alcohol hydroxy groups and secondary alcohol hydroxy groups [21,22]. COS can be structurally divided into two categories: homogeneous COS consisting of only GlcNAc or GlcN and heterogeneous COS consisting of GlcNAc and GlcN units [23]. It can be described and classified according to DP, molecular weight (MW), degree of acetylation (DA) and molecular weight distribution or polydispersity [24]. COSs with different structures may have different biological activities and functional mechanisms. The biological functions of COSs have been studied through heterogeneous and/or relatively poorly characterized mixtures of COSs, making it difficult to determine which molecules are responsible for the observed biological activities [25]. The molecular weight of COS is much lower than that of chitosan, which has better water solubility and has more physiological activities, such as antibacterial, antitumor, anti-inflammatory and lipid-lowering properties [26]. These physiological activities make COSs valuable and commercially promising food and pharmaceutical ingredients, which are used in many fields, such as medicine, functional food and agriculture. Some of the research hotspots can be found in Table 2.

Because sugars are so diverse in chemical structure, three-dimensional configuration and linkage, they can store orders of magnitude more biological information than DNA or proteins. However, the research and development of sugar compounds is still challenging because of the lag in the research on sugar chemistry and glycobiology. In recent years, polysaccharides from marine species have been increasingly investigated as gelling agents, drug candidates and drug carriers. Traditional methods cannot meet the needs of green development due to their low efficiency and high consumption [39]. At present, the enzymatic reaction can selectively form glycosidic bonds between saccharides to synthesize polysaccharides [40]. Among them, the enzymatic synthesis of glucan has achieved surprising results [41,42]. The synthesis of chitin has also been studied. Chitotetraose [(GlcNAc)_4_] was successfully synthesized using a chemical enzyme catalyzed by the *Bryum coronatum* inverting family GH19 chitinase BcChi-A mutant. Although there is certain potential in the synthesis of polysaccharides with specific structures, the current enzymatic synthesis of biomaterials is expensive, and large-scale synthesis is not economically feasible [43]. At the same time, many natural polysaccharides cannot be obtained by enzymatic synthesis due to the high requirements for donor saccharides. Compared with the limitations of enzymatic synthesis, enzymatic degradation does not require many synthetic steps, and the substrate is easy to obtain, which is more cost-effective. Enzymatic degradation of chitin may be a better strategy.

The classification, biochemical properties and catalytic mechanism of chitinase, chitinase deacetylase and chitosanase are reviewed. In addition, the design and modification of enzymes by artificial intelligence technology combined with protein engineering were proposed to solve the problems of stability and catalytic efficiency in the field of chitosan degrading enzymes. Finally, we prospected the recycling of enzymes and industrial production applications, hoping to contribute to the high value utilization of chitosan resources.

## 2. Chitinase

### 2.1. The Source and Biochemical Characteristics of Chitinase

Chitinase is a general term for enzymes that can specifically catalyze the breakdown of glycosidic bonds to degrade chitin into COS, chitosan biosaccharides or N-acetyl-d-glucosamine (GlcNAc). Chitinase is widely present in various organisms. Among them, chitinase derived from bacteria has attracted the most attention. A variety of bacteria from the ocean and soil can secrete different chitinases to degrade exogenous chitin. Chitinase has an important physiological function. In fungi, chitinase is the main component involved in remodeling the cell wall [44]. Arthropods use chitinase to regenerate and reconstruct the stratum corneum [45,46]. Bacteria obtain nutrition by secreting chitinase to degrade exogenous chitin [47], and plant chitinase plays a defensive role by targeting the cell walls of microbial pathogens [48]. In addition, the degradation of chitin by microbial chitinases has been an important research direction. In 2002, Sashiwa et al. [49] used a crude chitinase preparation from *Aeromonas hydrophila* H-2330 to hydrolyze α-chitin for the first time and obtained GlcNAc with a yield in the range of 66 to 77%. Research on chitin degradation by chitinase has developed rapidly. In 2011, Jamialahma K et al. [50] hydrolyzed α-chitin with crude enzyme from *Aeromonas* sp. PTCC for 24 h and obtained GlcNAc with a yield of up to 95%. However, an increasing number of studies have subsequently found that although the use of crude enzymes secreted by microorganisms to degrade chitin is simple and inexpensive, the amount of enzymes secreted by natural strains is unstable, the hydrolysis efficiency is low and the products are complex. At present, chitinase degradation by recombinant chitinase is a research hotspot. Chitinase with excellent properties from different microorganisms can be cloned and expressed by using genetic engineering technology [51].

The properties of chitinases from different sources are not completely the same and are generally reflected in the molecular weight, pH and temperature of the enzyme protein molecule. Table 3 shows that the molecular weight of most of the enzymes is approximately 20–100 kDa. In addition, Shehata An et al. [52] obtained a high-molecular-weight *Aspergillus griseoaurantiacus* KX010988 chitinase with a molecular weight of 130 kDa. The optimum temperature of most enzymes is between 40 and 60 °C, and the catalytic activity decreases significantly when the temperature increases. Some enzymes show thermal stability. Mohamed S et al. [53] obtained the acid-tolerant and heat-stable endochitinase CHIA-MT45 from *Melghiribacillus thermohalophilus* strain Nari2A^T^. The optimum temperature is 90 °C at pH 3.5 and in the presence of 2 mM Ca^2+^, and enzyme activity can be detected over a wide temperature range of 40–100 °C. The optimal pH value of most chitinase activities is between 4 and 8, while some enzymes have optimal activity under strong acidic conditions [54], and some enzymes have optimal activity at a pH of 11 [55]. Different metal ions have different effects in different bacterial genera. For example, Mn^2+^, Co^2+^ and Mg^2+^ are generally chitinase activators. Cu^2+^ is an activator of ChiA-Hh59 chitinase [56] and an inhibitor of SaChiA4 chitinase [57]. The different effects of metal ions may be closely related to the conformational changes of the enzyme. We can obtain a chitinase with optimal activity according to the properties of the enzyme to exert its physiological function.

### 2.2. The Structure and Catalytic Mechanism of Chitinase

Chitinases can be divided into endochitinases (EC 3.2.1.14) and exochitinases (EC 3.2.1.29). Endochitinases can be subdivided into two subgroups. One type is chitobiosidase, which cleaves the diacetyl group at the nonreducing end of the chitin chain in units of dimers; the other type is β-(1,4)-N-acetylglucosaminidase (NAGase, EC 3.2.1.30), which trims the nonreducing end of chitin to produce N-acetylglucosaminidase. Endochitinase is the main research object and can be divided into chitinase GH18 and GH19 families [51]. At present, research is mostly focused on chitinase GH18 family members, and the structure and catalytic mechanism of chitinases will be analyzed from the perspective of endolitic chitinases.

In general, GH18 chitinase has only one catalytic domain, which consists of classical TIM barrel folds with the highly conserved characteristic sequence DXDXE. The core region of the TIM barrel-like fold structure is composed of eight β-strands and eight α-helices, forming (β/α) eight barrel-like folds. The characteristic motif DXDXE is located in the ring between β4 and α4 and contains glutamate catalytic acid–base residues [71]. TIM barrel domains have few sequence characteristics and many functions. The eight rings at the carboxyl terminus of the β-chain determine the specificity of the enzyme [72]. With the progress in enzyme engineering, there have been reports of chitinases with two GH18 family catalytic domains. Chitinases with dual catalytic domains usually have different catalytic activities. Larsbrink et al. showed that chitinase ChiA from Flavobacterium Johnsoniae contained two GH18 domains, which had endosomal and exosomal activity, respectively [73].

**Figure 2:**
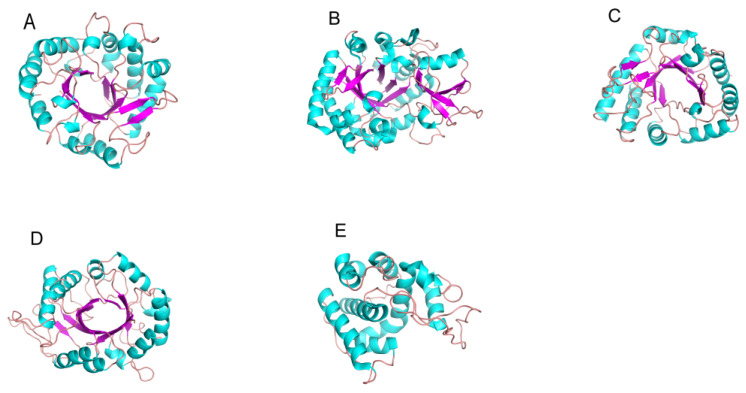
Three-dimensional structures of chitinase: (**A**) Chitinase Chi23 (PDB ID: 6K7Z) of family GH18 from *Pseudoalteromonas aurantia;* (**B**) Chitinase ChtII (PDB ID: 5Y29) of family GH18 from the insect pest *Ostrinia furnacalis*; (**C**) Chitinase PrChiA-cat (PDB ID: 4RL3) of family GH18 from the *fern, Pteris ryukyuensis;* (**D**) Chitinase ChiA1 (PDB ID: 2XVP) of family GH18 from *Aspergillus fumigatus;* (**E**) Chitinase ChitA (PDB ID: 4MCK) of family GH19 from Zea mays. β-Strands are shown in cyan-blue and α-helices in magenta. Loops and other secondary structures are in ginger.

The GH18 chitinase is divided into three subfamilies, known as ChiA, ChiB and ChiC. The major difference between the three enzymes is that the catalytic domain of ChiA has more insertion domains than those of ChiB and ChiC. The most studied enzyme is ChiA, whose TIM barrel domain contains the β7 and α7 insertion domains (CIDs) connecting the core domain, which contains 5-to-6 antiparallel β-chains and 1-to-2 short α-helices [71]. The CID and TIM barrel-shaped substrate-binding clefts are arranged side by side to form a wall to increase the cleft depth in the cleft-like binding site, which provides deep substrate-binding clefts that can bind to long-chain substrates [74]. This insertion of components into the catalytic domain provides a specific binding site or remodeling of the active site, enabling the enzyme to recognize substrates of different shapes and sizes and thus exert substrate specificity. The substrate binding groove of GH18 chitinase from different sources is different. The chitinase obtained from bacteria has a deep substrate binding groove containing a large number of aromatic residues [75] (Figure 2A), while the substrate-binding cleft of chitinase obtained from insects is long and deep [76] (Figure 2B), and the chitinase obtained from plants and fungi has a shallow substrate-binding groove [77,78,79,80,81] (Figure 2C,D).

**Figure 3:**
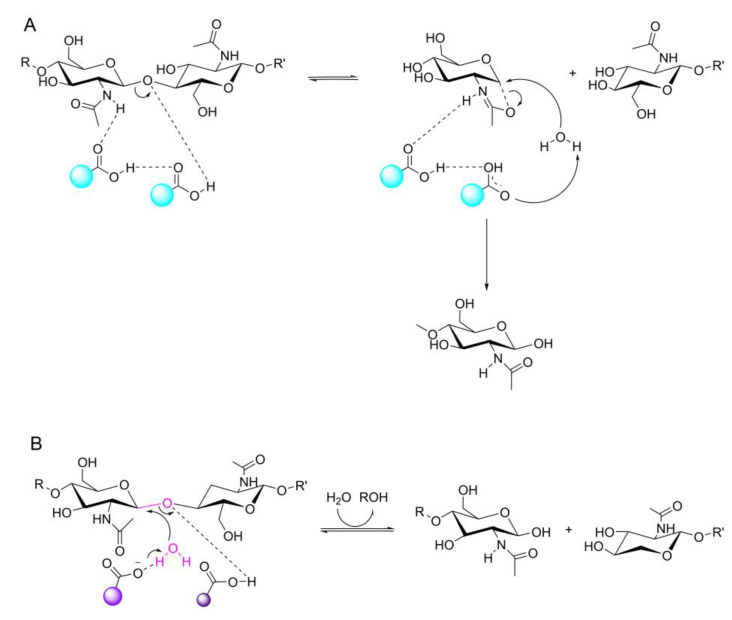
(**A**) The substrate assisted mechanism of chitinase. Acid/base amino acid group are in red ball. (**B**) Single displacement catalytic mechanism of chitinase. Base amino acid group is in bright purple ball. Acid amino acid group is in dark purple ball.

The GH18 family catalyzes the hydrolysis reaction through a substrate-assisted mechanism [74] (Figure 3A). In the catalytic process of chitin, the subsites +1 and +2 of the enzyme interact with the reducing end of the chitin substrate, and hydrolysis occurs in the binding cleft between the subsites −1 and +1 [74]. The Brownian motion of the enzyme along the chitin chain is positively modified by a substrate-assisted mechanism. The asymmetric subsite structure determines the direction and degree of the progressive degradation of crystalline chitin [82]. During the degradation catalyzed by GH18 chitinase, the enzyme is first adsorbed on the substrate surface, and then the polysaccharide chain binds to the active site of the catalytic module. In the substrate-assisted catalytic mechanism, the catalytic amino acid residue and chitin N-acetyl group first trim the glycosidic bond without attacking the water, and then the water molecule approaches the catalytic center. During the reaction, the free N-acetylamino group of the substrate acts as the internal nucleophile, and the second aspartic acid in DXDXE hydrolyzes the oxazoline ion formed by N-acetylamino deprotonation or the oxazoline intermediate. With the hydrolysis of the intermediate product, the glycosidic bond is broken, the product chitosan is released from the chain end, and the next chitosan unit is stripped from the crystal surface along with the previous step [82]. N-acetyl groups in oligosaccharides accelerate the cleavage of glycosidic bonds. The catalytic assembly then slides along the polymer substrate to the next cleavage site. The decrystallization of a single chain from the crystal surface is the rate-limiting step of the enzyme degradation reaction.

There are few studies on GH19 chitinase at present. The structure of GH19 chitinase is rich in α-helix, and there is a high structural similarity between the enzymes, which use a single displacement catalytic mechanism (Figure 3B). Some GH19 chitinases have a six-ring structure, which is responsible for substrate binding at both ends of the substrate-binding groove, and the enzyme catalytic center is located in the middle of the substrate-binding groove [83]. Kawamoto D et al. have shown that the flexible loop structure (loop III) at the end of the COS binding groove is not directly involved in enzymatic activity, but controls protein stability and enzymatic properties through core functional regions [84]. In an enzyme without the ring structure, the substrate-binding groove is shorter than that of an enzyme with the ring structure [83] (Figure 2E). Generally, the domains of GH19 chitinase include the N-terminal signal peptide region (SP), catalytic region (CCD), chitin-binding region (CBD) and C-terminal extension region [85]. GH19 chitinase has a unique subsite preference and high specificity and can control the substrate hydrolysis process to produce oligomers with more specific degrees of polymerization (DPs).

## 3. CDA

### 3.1. The Source and Biochemical Characteristics of CDA

Since Araki Y and Ito E [86] discovered CDA from *Mucor rouxii* in 1974, an increasing number of CDAs have been discovered. Although the sources of CDA are abundant, the yield of naturally obtained CDA is low, and the crude enzyme solution has many impurities and is not easy to separate and purify to obtain the target CDA. Although the production of CDA-producing strains can be improved by mutagenesis, the mutagenized strains are usually unstable. These problems can be solved by genetic engineering technology through a series of genetic modifications to obtain enzymatic characteristics and a stable yield of CDA. At present, many engineered bacteria have been successfully constructed and expressed in a variety of hosts, but most of them are *Escherichia coli*. CDA has different biological functions and can be used to destroy the internal barrier of the epidermis of *Lasioderma serricorne* to achieve pest control by taking advantage of the key role of CDA in *L. serricorne* molting and wing development [87]. It is possible to kill *Magnaporthe oryzae* by taking advantage of the fact that the coding gene of CDA is an essential gene for appressorium differentiation of the fungal infection structure [88]. CDA can be used to remove the acetyl group to convert (GlcNAc)*_n_* to N-glucosamine (GlcN)*_n_* [89,90].

CDAs from different sources have great differences in molecular weight, optimal temperature and pH. The physicochemical properties of the CDAs from some microbial sources are shown in Table 4. The molecular weight of most CDAs ranges from 40 to 150 kDa, the optimal pH value ranges from 5.5 to 9.0 and the optimal temperature is mostly 50 °C. Different kinds or concentrations of metal ions have different effects on the activity of CDA; for example, Ca^2+^ shows activation and inhibition effects on different enzymes [91,92]. The CDA obtained from *Aspergillus comorbifera* strains with higher enzyme activity [93,94] is an acidic glycoprotein with high thermal stability. The optimal pH is 7.0, and the CDA has high stability at pH 4.0 to 7.5. The optimal temperature is 50 °C, and it remains active even at 30–100 °C for 1 h with a wide range of substrates. These include glycol chitin, oligosaccharide acetylglucosamine, smaller lengths of chitin, colloidal chitin, carboxymethyl chitin and other substances containing amino groups. The conditions with the highest CDA enzyme activity are mild and do not require harsh conditions.

### 3.2. The Structure and Catalytic Mechanism of CDA

Chitin deacetylase (EC 3.5.1.41) belongs to the CE4 enzyme family [106]. A common feature of the CE4 family is that it has the same primary protein structure. Since its conserved region is similar to the conserved gene fragment of the NodB protein, it is called the NodB homologous domain [107]. This region is characterized by (β\α) eight-barrel folds, with a series of rings decorated by the β barrel forming the binding pockets of most carbohydrates that specifically bind to the residues of GlcNAc at the nonreducing end of acetylated chitin.

**Figure 4:**
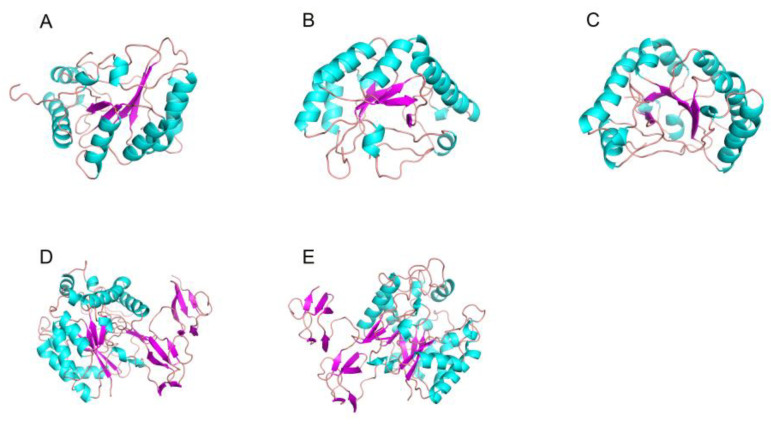
Three-dimensional structures of CDA: (**A**) *Cl*CDA (PDB ID: 2IW0) from the fungal pathogen *Colletotrichum lindemuthianum*; (**B**) *Ar*CDA (PDB ID: 5LFZ) from a *marine Arthrobacter species*; (**C**) *An*CDA (PDB ID: 2Y8U) from *Aspergillus Nidulans Fgsc* A4; (**D**) *Vc*CDA (PDB ID: 4NY2) from the Vibrio cholerae; (**E**) *Vp*CDA (PDB ID: 3WX7) from *Vibrio parahaemolyticus*. β-Strands are shown in cyan-blue and α-helices in magenta. Loops and other secondary structures are in ginger.

Among the known CDA crystal structures, CICDA, AnCDA and ArCDA all have compact catalytic domains [94,108,109] (Figure 4A–C), similar to the denatured (β\α) eight barrels of TIM barrels. Different subsites form significantly different rings around the substrate-binding sites, and the shorter ring can form relatively open substrate-binding clefts. When the ring is conformational, it can trap the substrate in the substrate-binding pocket. The catalytic domains of *Vc*CDA and *Vp*CDA consist of deformed (β/α) seven barrels, lacking a β/α repeat sequence of conventional TIM barrels, and consist of seven parallel double chains in the center, forming a highly twisted β-barrel surrounded by α-helices with nearly identical substrate-binding pockets [110,111] (Figure 4D,E).

**Figure 5:**
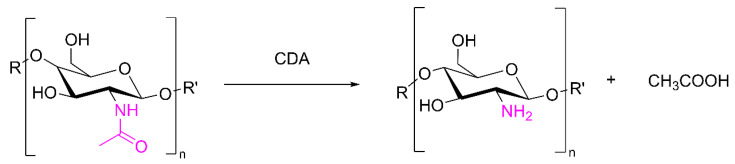
The catalytic reaction of CDA.

The enzymatic reaction of CDA directly shears the amide bond on the chitin molecule and reduces the acetyl degree of chitin without breaking the chitin chain (Figure 5). The crystallinity of chitin affects the deacetylation rate of CDA. Under general conditions, the crystal fibrous degree of small-molecule chitin is much smaller than that of large-molecule chitin, and the crystal gap is larger, which is conducive to CDA entering the interior of chitin. CDA has no activity against COSs with fewer than three sugar units. The substrate bias and deacetylation patterns of CDA from different sources are also significantly different.

There are three CDA enzymatic hydrolysis modes, namely, the multiple attack mode, the multichain mode and the single-chain mode [112,113,114,115]. All three modes are metal-assisted through an acid/base catalysis mechanism. Block copolymers are obtained by the multichain attack and single-chain modes, while randomly distributed binary heteropolysaccharides are obtained in multichain mode. In the multichain mode, the product is a mixture that can even control the enzymatic process; therefore, the deacetylation process of each enzyme with multi-chain mode must be carefully studied [116]. The deacetylation patterns of different CDA groups are controlled by key loops, and the substrate specificity of the enzyme is determined by the different key loops in the catalytic center. In view of the specific deacetylation mode of CDA, some researchers have proposed [110] a “substrate capping model” (or alternative covering model). Enzyme key rings cover the edges of the clefts in the substrate combination, can alter the conformation and differences in blocking of the enzyme CE4 combination cleft can access the site, namely, the combination of the substrate along the enzyme cleft slides in different ways, and the ability to bind to the enzyme is dependent on whether the substrate GlcNAc residues can be exposed to the enzyme catalytic sites, which achieved results in the removal of acetylation. In addition, these rings can be dynamically tuned to create new orthosubunits that allow enzymes to bind to longer oligosaccharide chains. With the further study of chitin deacetylase, it is possible to use the region of chitin deacetylase to selectively produce sequentially determined heterogeneous COS [116].

## 4. Chitosanase

### 4.1. The Source and Biochemical Characteristics of Chitosanase

Since Monaghan R et al. [117] first proposed chitosanase in 1973, an increasing number of researchers have successively discovered chitosanase from a variety of microorganisms. Microbial chitosanases in nature have different biological activities. Microorganisms hydrolyze chitosan to obtain nutrients via chitosanase secreted outside the cell [118]. Chitosan is hydrolyzed industrially by chitosanase to produce COS [119], which can also be used as an ecofriendly biological control reagent for the control of plant pathogens and diseases and insect pests [120,121]. Therefore, chitosanase with high enzyme activity can be obtained by selecting different enzyme-producing microorganisms through biotechnology.

The basic biochemical properties of chitosanase from different sources are also different. Table 5 shows that the molecular weight of chitosanase is between 20 and 70 kDa, and Song Ys et al. [122] obtained a chitosanase with a lower molecular weight. The molecular weight of the chitosanase GH8 family is larger than that of the chitosanase GH46 and GH75 families. The essential groups of the enzyme active site are affected by the environmental pH, and the optimal pH range of most chitosan activities is between 4 and 8. Because chitosan is insoluble in alkaline solution, the enzyme activity decreases obviously when the pH is higher than 7. Liang TW et al. [123] obtained a novel amphoteric chitosanase, CS038, from *Bacillus fungoides* TKU038. The optimal pH values were 6 and 10, and CS038 showed 100% and 94% chitosan degradation activity, respectively. The spatial structure of the enzyme was affected by temperature, and the optimum temperature for most of the chitosanase activity was mainly in the range of 30 to 60 °C. In particular, Qin Z et al. [124] identified GSCSN46A, a novel cold-adapted chitosanase from *Gynuella sunshinyii*. The optimal activity of the enzyme was 30 °C, and the enzyme maintained 70% activity even at 15 °C. In general, heavy metal ions have a serious inhibitory effect on enzyme activity and even completely inactivate enzymes, such as Cu^2+^, Hg^2+^ and Co^2+^. Inorganic ions such as Mg^2+^, Ca^2+^ and Mn^2+^ can increase enzyme activity.

### 4.2. The Structure and Catalytic Mechanism of Chitosanase

Chitosanase belongs to the glycoside hydrolase family. According to the mode of action, chitosanases can be divided into endochitosanases (EC 3.2.1.132) and exochitosanases (EC 3.2.1.165), in which endochitosanases randomly cut β-(1,4) glycosidic bonds in the reducing end of the chitosan chain to produce COSs with different degrees of polymerization, and exochitosanases produce GlcN monosaccharides by breaking the glycosidic bond in the nonreducing end of the chitosan chain. The acetylation pattern of chitosan and the subsite specificity of the enzyme determine the difficulty of chitosan degradation and the product type [137]. Each subsite in the substrate-binding cleft of the enzyme binds to a sugar monomer subunit, thus showing a preference for GlcN and GlcNAc.

According to the classification system of the Carbohydrate-Active enZYmes Database, chitanases are divided into seven GH families: GH3, GH5, GH7, GH8, GH46, GH75 and GH80 (Carbohydrate-Active enZYmes Database, http://www.cazy.org/, accessed on 21 June 2021). All of these families of chitosanases have the ability to cleave the partially acetylated GlcN–GlcN bond in chitosan. Among them, the GH46, GH75 and GH80 families contain only chitosanases, while the other four families also have substrate-specific enzymes other than chitosanases [138].

**Figure 6:**
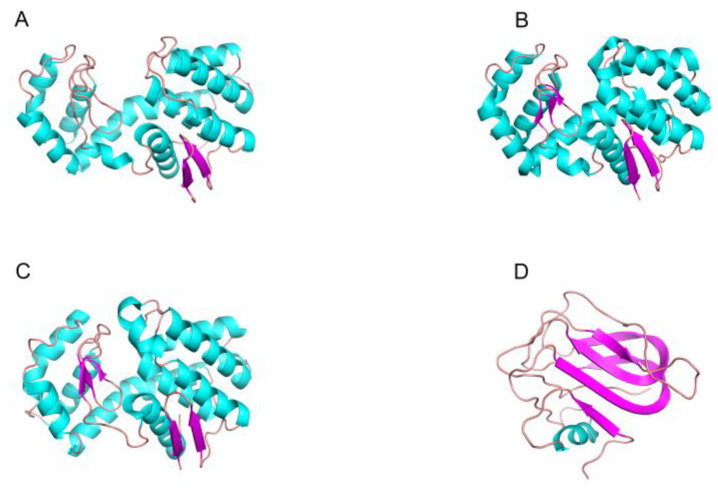
Three-dimensional structures of chitosanase: (**A**) Chitosanase (PDB ID: 1CHK) from *streptomyces* N174; (**B**) chitosanase OU01 (PDB ID: 4OLT) of family GH46 from *Microbacterium* sp.; (**C**) chitosanase CsnMY002 (PDB ID: 7C6C) from *Bacillus subtilis*; (**D**) CBM32 carbohydrate-binding modules (PDB ID: 2RVA) from a *Paenibacillus* sp. IK-5. β-Strands are shown in cyan-blue and α-helices in magenta. Loops and other secondary structures are in ginger.

Among them, GH46 chitosanase has good enzymatic hydrolysis characteristics and has been extensively studied in terms of its enzymatic mechanism and protein structure. Most GH46 family chitosanases are found in bacteria and have a typical α-helical fold structure in which a negatively charged substrate-binding cleft exists between two spherical lobules in the three-dimensional structure of the enzyme. There are two conserved acidic catalytic residues involved in the catalytic reaction: Glu, which provides protons as a total acid, and Asp, which serves as a total base [139,140] (Figure 6A). Lyu Q et al. [141] first proposed the binding mechanism between the (GlcN)_6_ substrate and chitosanase, and the enzyme recognizes the substrate through key residues. Several residues in the binding cleft (Ser27, Tyr37, Arg45, Thr58, Asp60, His203 and Asp235) play an important role in the interaction between the enzyme and the substrate, and the interaction between the pyranose ring of the substrate and the substrate-binding region of chitosanase further stabilizes the substrate–enzyme complex [142] (Figure 6B). The type of chitosanase has a significant effect on the way that the substrate acts.

**Figure 7:**
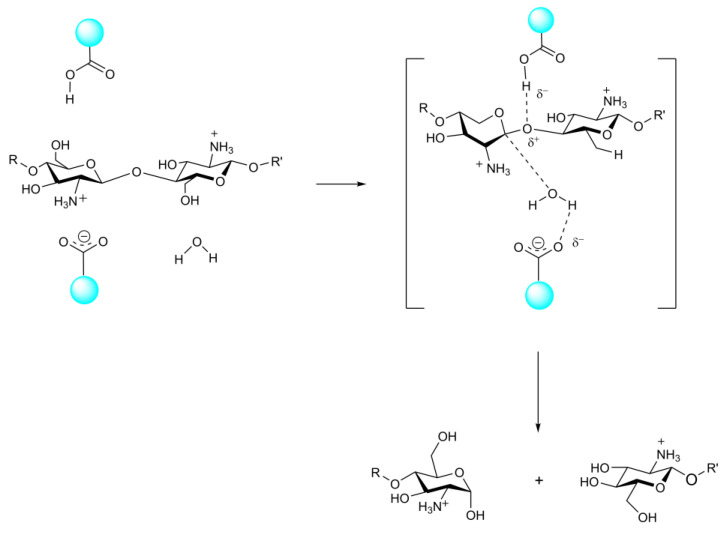
The reversal mechanism of GH46 chitosanase. Acid/base amino acid group are in cyan-blue ball.

GH46 chitosanase is a nonprocessing endonuclease, and its enzymatic mechanism is a reversal mechanism that can change the conformation of isomers in the chitosan unit (Figure 7). The CSNMY002 enzyme [143] has a closed tunnel with a size of ~4 × 6.5 × 8 Å in the substrate-binding region. When it binds to (GlcN)_6_, (GlcN)_6_ is located in the active center. After (GlcN)_6_ enters the tunneled substrate-binding region and degrades, the cleft of the polymer substrate reduces the total binding energy of the enzyme–substrate complex. The cleaved substrate is dissociated from the enzyme, and the enzyme continues to bind to the substrate until the final product is produced [143] (Figure 6C). However, the mechanism of how the substrate enters the substrate-binding region of the tunnel is unclear. Artificial and natural chitosan are amorphous and heterogeneous polysaccharides containing GlcN and GlcNAC residues. A chitosan-specific noncatalytic carbohydrate-binding module (CBM) has been identified. In the degradation of insoluble polysaccharides, the CBM may promote the binding of enzymes to substrates by increasing the concentration of additional enzymes near the substrates [144]. In the CBM32 family, tandem DD1/DD2 protein pairs (GlcN)_6_ are highly specific, and a single CBM can accommodate at least two GlcN units in the loop extruded from the core β sandwich structure. The synergistic effect of the two CBMs promotes the degradation of chitosan, suggesting that CBMs may assist chitosanase by introducing the chitosan chain into the catalytic cleft [145,146] (Figure 6D).

## 5. Design and Modification of Enzyme

The unique composition and structural characteristics of each thermophilic and thermophilic enzyme determine its stability. Compared with cold-adapted enzymes, thermophilic enzymes have higher stability due to their more rigid structures. While proteins are denatured at extreme low temperatures, the current mechanism for the phenomenon of cold denaturation involves the interaction of water molecules with buried hydrophobic and/or surface polar residues [147]. Heat-stable proteins were also found to be more resistant to cold denaturation. The thermal stability of chitinases has been widely considered. The enzymatic hydrolysis reaction process at high temperature not only increases the solubility of the substrate and product but also reduces the viscosity of the solution. There are two main sources of thermophilic enzymes: one is to screen thermophilic microorganisms in a high-temperature environment to produce enzymes with good stability; the other is to improve the thermal stability of normal-temperature enzymes by using gene engineering and protein engineering technology. However, in the former method, thermophilic microorganisms require harsh culture conditions and complex acquisition processes. ChiEn3 from *Coprinopsis cinerea* is the first reported thermophilic chitinase from a nonthermophilic fungal ash enzyme, also belonging to the GH18 chitinase family. ChiEn3 has clear tunnel-like substrate-binding clefts and has extremely high hydrolytic activity for commercial 85% deacetylated chitosan, which has the advantage of preparing COS [86,141]. In the future, we need to be good at using genetic engineering and protein engineering technology to improve the kinetic stability of enzymes, such as the optimal temperature, half-inactivation temperature and half-life, so that they can maintain their optimal enzymatic function for a long time.

At present, the chitooligosaccharide with DP or mixture is mainly studied, which is difficult to determine which molecules are responsible for the observed biological effects. Some studies start from the reaction conditions, and the enzyme is first involved in the reaction in the form of immobilization so that the enzyme can be continuously produced. Secondly, chitooligosaccharides with different DP range were obtained by controlling the reaction conditions. The immobilization of enzyme has a great relationship with carbohydrate binding module, and a new type of recombinant enzyme was formed by fusion of natural chitosan enzyme and CBM [148,149]. This domain provided the fusion protein with high specific binding ability on the gel polysaccharide carrier and improved the stability of the enzyme. The chitosanase immobilized gel polysaccharide packed bed reactor (CICPR) was constructed, and the different reaction conditions (such as the amount of chitosanase, flow rate and substrate concentration) were optimized. The enzymatic hydrolysis process was controllable, which provided the potential for industrial scale stability and reproducible preparation of chito-oligosaccharides with different DPs. In recent strategies to improve the long-term stability and reusability of enzymes, Mena-Giraldo P et al. [150]. immobilized enzyme proteins in a new photosensitive polymer Janus micromotor (JM) based on accelerating diffusion-mediated enzyme–substrate interaction, which absorbs ultraviolet light, protects enzyme activity through magnetic/catalytic movement and accelerates enzyme–substrate degradation. Chen Y et al. [151] expressed and immobilized specific GH8 family CHI-1 enzyme on the surface of Escherichia coli BL21 by cell-surface display technology and successfully produced COS with value-added effects by recombinant DNA technology.

Through retrieval, it was found that the current stability and catalytic efficiency of specific enzymes have not yet reached the requirements of mature process conditions. In view of these situations, in addition to the use of computer-aided analysis of candidate enzymes in the protein database and reasonable and reliable prediction of their substrate specificity and activity, modern protein engineering tools can also be used to directly introduce the required characteristics into the target site to synthesize suitable new enzymes [152]. These technologies benefit from the application of artificial intelligence and high-throughput sequencing and screening technologies.

Through the modification of natural enzymes, the limitation of understanding the relationship between protein sequence and structure was broken. The re-design of enzyme is based on the natural enzyme skeleton, and the use of computer intervention is more and more used in the transformation of enzyme production. The design of enzymes is very challenging because of their own complexity, especially the analysis and understanding of enzymes in nature to find suitable biological catalysts. Scientists also hope to use ab initio design methods, based on guiding the physical principles of protein folding to explore the full sequence of protein space design does not exist in nature enzyme proteins. Metalloenzymes account for almost half of the natural enzymes and are also hot spots designed from scratch. With the innovation of science and technology, the method of artificial intelligence to design enzymes has also been studied. With the aid of AI, directed evolution expands the sampling space of protein sequences. The directed evolution of enzymes first introduced random mutations into the genes of enzymes to construct a random mutation library; then, the enzyme protein mutants with improved characteristics were directional screened; finally, the genes extracted from the mutants were subjected to a new round of random mutation until the target enzyme mutant was found. Directed evolution does not require detailed basic physical or biological pathway models to produce suitable variants that enable biocatalysis to reach production scale successfully. Reliable machine learning methods are also the starting point of artificial intelligence enzyme design. Different from the traditional computer design method, the machine can find the best folding way of protein sequence through deep learning, and can predict the structure and function of protein. At present, the technology of high-throughput sequencing is becoming more and more perfect, which can obtain quite optimistic high-quality enzyme molecules.

In the glycosidase family, genes encoding GH1 β-glucosidase (TaBgl2) have been isolated and characterized by relevant computer analysis, and the function of recombinant enzymes has been predicted [153]. The dominant mutant M137E/N269G was obtained by directed evolution, and the xylanase with high catalytic efficiency and good thermal stability was obtained [154]. The thermal stability of the GH11 family xylanase mutant Xyn372 obtained through directional evolution and rational design was improved [155]. The enzyme structure was rationally designed, and the structure of Mut-2-8 bound to (GluNAc)2 has two acetyl groups bound to the catalytic region and six hydrogen bonds, improving the catalytic activity and performance of Mut-2-8 [156]. The structure–function relationship of the residues related to the thermal stability of GH45 endoglucanase was also studied. The biochemical properties of the double mutant T90A/Y173F of β-1,4-endoglucanase CTendo45 were improved after the conservative non-catalytic residues and N-glycosylation sites were optimized [157]. Su H et al. cloned a new and a second GH5 chitosanase OUc-Csngly from *Streptomyces bacillaris*. Molecular docking analysis showed that the c-2 sugar unit affects the binding of the enzyme to oligosaccharides, cutting any glycoside bonds of the identified chitosan substrate in a random endotangent mode [158].

## 6. Concluding Remarks and Future Directions

The research field of natural compound resources is emerging continuously and has great potential in terms of its biological activity. Chitosan and its derivatives as natural compounds have wide potential in medicine and food. Enzymes are key participants in the process of the production of chitosan and its derivatives because of their unique characteristics. It is hoped that these characteristics and mechanisms can be used as references for the identification of novel enzymes.

In the chemotaxis theory of enzymes, an increase in substrate concentration can increase the diffusion coefficient of enzymes [159]. At the same time, enzyme catalysis can also improve its effective diffusion rate, making it possible for the enzyme to efficiently degrade high concentrations of substrate [160]. Preparation of chitosan and its derivatives in industry needs efficient stable enzymes to ensure effective bioconversion, but currently in the enzymatic preparation of chitosan oligosaccharides, the corresponding enzyme speed is slow, the ability to reduce the viscosity of the polymer solution after reducing the concentration of substrate needs a large-volume reaction system and the amount of enzyme needed is great. The accuracy and effectiveness of enzyme catalysis are often unable to meet the requirements of industrial applications and lack catalytic functions with commercial value. The crystallinity of chitin must be pretreated by chemical, physical or other methods before it can be effectively degraded further by chitinase. The challenges of high complexity and high pretreatment costs can be overcome if chitinase can directly hydrolyze chitin powders at industrial rates. Compared with the synergistic effect of ChBD ChiA1 in the original chitinase Al, engineered ChBD produced excellent effects on insoluble chitin substrates, which was a very important step towards large-scale processing of chitin and had great economic benefits [161]. The fermentation medium of fecal bacteria, Alca F2018, proposed that the recycling of RSC to produce chitosan can ignore the purification step and obtain chitosan with high yield and sufficient degree of deacetylation and low crystallinity, which is an environmentally friendly process. Therefore, the large-scale fermentation process for chitosan production can make up for the shortcomings of chemical and purifying enzymatic methods [162]. Michal B Kaczmarek et al. synthesized a eukaryotic polycistronic expression system, which uses the de-degenerate nature of the virus’s self-processing sequence and genetic code to simultaneously express three genes encoding proteins with chitin and chitosan decomposition activities under the control of a promoter. The multistage separation process of biocatalyst, such as deacetylation and depolymerization, can be carried out in a coherent operation, and the cost of the process can be significantly reduced [163]. The complex structure of chitosan requires high substrate selectivity for its degrading enzymes. At present, the products prepared by enzymatic hydrolysis are mostly oligosaccharides with an uncontrollable degree of polymerization. The preparation of oligosaccharides with a single degree of polymerization is still a major challenge, especially to study the basic structure–functional relationships of oligosaccharides that need to be clearly defined. To solve the above problems, in addition to identifying new hydrolases suitable for industrial application, we can also use protein engineering to conduct directed evolution and design recombinant enzyme genes to obtain enzymes with high activity and high stability.

The hydroxy group in each sugar residue can be independently linked to another carbohydrate, indicating that the composition and structure of polysaccharides are relatively complex. However, with the development of protein engineering, the molecular weight and acetylation degree of oligosaccharides have been controlled at different levels. Chitosan can be degraded by several degradation mechanisms, including most commonly hydrolysis by glycoside hydrolases with endonucleases and exonucleases and lysis by oxidoreductases. To explore more complex utilization and transformation of enzyme degradation products, separation and purification of products should be performed. Enzymes are potential immune modulators [164], and the presence of enzyme proteins may cause human autoimmune reactions, so it is necessary to remove the enzyme proteins in the enzymatic hydrolysates in time. On the other hand, COSs with different degrees of polymerization have different physiological functions, so it is of great significance to isolate and purify single COSs with specific characteristics. As different separation technologies are used in the separation of chitosaccharides, commonly used separation methods include size exclusion chromatography (SEC), metal affinity chromatography and ion exchange chromatography [165]. It is a potential method to separate single chitooligosaccharides with molecular weight determined by advanced preparative chromatography. A novel GH75 family chitosanase was identified from *Penicillium oxalate* M2 based on a new screening strategy. A 19.34 times purification was achieved on a cation exchange column, and the resulting enzyme showed strict specificity for chitosan. The final product of hydrolysis is chitosaccharide with polymerization degree 2–5, without glucosamine or acetylglucosamine [136]. In addition, the immobilized enzyme can also separate the enzyme from the product and reuse it, which is easier to operate than the separation and purification of chitosan oligomer prepared by free enzyme.

Enzymes acting on the chitin polysaccharides mentioned above can be combined with other enzymes that promote enzymatic hydrolysis. Lycolytic polysaccharide monooxygenase (LPMO) is a newly discovered enzyme tightly bound to copper ions, which can cut the polysaccharide chain through oxidation in the crystalline region of the refractory polysaccharide [166,167]. LPMO is classified as ‘auxiliary activity (AA)’ in the Carbohydrate-Active Enzymes (CAZy) database [168]. Studies have shown that AA10, AA11 and AA15 have chitin degradation activity. A new type of AA11 family protein, Tg AA11, has a considerable synergistic effect on the degradation of chitin by highly efficient commercial chitinolytic enzyme mixture (Sg-chi). In the presence of Tg AA11, the degradation of α-chi and β-chi increased by 39.9% and 288.2%, respectively [169]. Obviously, LPMOs played an important role in enhancing chitin-related specific enzyme degradation.

Chitosan oligomers are important natural resources and have great potential application in industrial production because of their various biological activities. However, the industrial preparation method is still a chemical method, and the commercial application of enzymes is still a difficult and complex process. As a biocatalyst, there is little demand for protective groups due to the excellent regional selectivity when enzymes bind to substrates. In addition, the enzyme is biodegradable, not only reducing the waste generation, and is harmless to the environment, which is better than chemical catalysts. At the same time, the reaction conditions required for the biological catalytic reaction are relatively mild, and no special solvent is needed, so it has higher safety. The biological function of the enzyme is determined by its three-dimensional structure to a certain extent. Structural analysis and prediction are important ways to understand the function of the enzyme. This paper introduces the biochemical characteristics, structure and catalytic properties of the enzyme, hoping to help find and design the enzyme with high catalytic effect and industrial application potential. We look forward to the creation of a new, clean and green industrial production mode.

## Figures and Tables

**Table 1:** Recent applications of chitosan.

Name	Physical Representation	Function	Mechanism of Action	Refs.
CTS	DD: 92.78% MW: 46.33 kDa	Antifungal applications against human pathogens.	The increased cationic charges on the nanoparticle surfaces that may contribute to enhanced interaction with the negatively charged cell membrane and its disruption.	[11]
CTS	S@CS NPs were prepared by mixing the chitosan (CS) and spike protein (S), (CS: 5 μg, S: 5 μg)	Favorable mucosal vaccine adjuvant with aerosol inhalation	The CS-mediated inhalable nanovaccine stimulated balanced immunity between humoral and cellular immunity without systemic toxicity	[12]
CTS	DD: 77.6–82.5% viscosity: 751–1250 mPas (1% in 1% acetic acid, 20 °C)	Used as the polymer basis of the film	The film releases the drug along a saturation curve, initially faster for the anionic drug and slower for the cationic drug.	[13]
CTS	MW: 50–190 kDa	As an injectable delivery system	Promoting the change of surface charge from negative to positive and to enhance their interaction with cells	[14]
CTS	NA	Drug delivery system	Act as a barrier material to delay the diffusion and degradation of PLGA microspheres for longer duration of action.	[15]
OCS	Mn ≤ 3000 Da	Bone regenerative properties are prepared using sodium tripolyphosphate (TPP) as a crosslinker	Promote osteogenesis with its anti-inflammatory and antioxidant abilities	[16]
CS	DD ≥ 95%	High-performance protein-based multifunctional adhesives	When CS molecules and the fractured BN came into close contact, they reacted with each other and formed a high interfacial binding energy	[17]
CS	MW: 50–90 kDa DD: 75–85%	As nanofillers	Significant improvement in surface hydrophobicity, moisture and light barrier potential, mechanical strength and antioxidant properties of the composite films	[18]
CS	MW: 20 kDa DD: 90%	The natural polymeric flocculants	Restrained the release of organic substrates from solid phase to liquid phase, from macromolecules to micromolecules and finally to methane	[19]
CS	DD: 90% MW: 3 and 10 kDa	Permeation enhancer	Carries a positive charge and can increase skin permeability by opening the tight junctions of the stratum corneum	[20]

NA: Not available; CTS: Chitosan; CS: Chitosan; OCS: Chitosan; MW: Weight-average Molecular Weight; DD: Degree of Deacetylation; Mn: Number-average Molecular Weight.

**Table 2:** Recent applications of COS.

Name	Physical Representation	Function	Mechanism of Action	Refs.
SCOS	DP: 3–7 MW: 2 kDa sulfate content: 30%	Enhance the anti-influenza A virus (IAV) activity of COS	Blocked IAV entry through interfering with both virus adsorption and membrane fusion processes	[27]
COS	DP: 3–7 MW ≈ 1 kDa DD: 98.69%	Non-toxic biological antibacterial agent	Inactivated Escherichia coli through the sublethal injury process. For Staphylococcus aureus, some cells were induced into VBNC state by COS	[28]
EVs-COS	NA	As a scaffold to promote the effects of AMSC-derived	EVs-COS could facilitate cartilage injury repair and have better protective effects on OA by promoting the viability and migration of chondrocytes, suppressing cell apoptosis and regulating COL1A1, COL2A1, OCN, OPN, RUNX2, c-Myc, p53, Bcl2 and the Akt/PI3K pathway.	[29]
COST/COSM	COST (MW ≤ 1000 Da) COSM (MW ≤ 3000 Da)	Ameliorate APAP-induced liver oxidative damage	Inhibit toxic APAP metabolism, inhibit oxidative damage and the apoptosis pathway, increase activation of the liver antioxidant pathway	[30]
COS	DD: 95.6% high purity ≥ 90% DP: 3–6	Antifungal activity	Inhibitory to food spoilage fungi via damaging cell walls and membranes and disrupting normal cellular metabolism.	[31]
COS	MW < 1kDa DD: 88% DP: 2–6	Alleviate the symptoms of constipation by beneficially regulating the levels of endogenous metabolites.	Most significantly changed metabolic pathways in plasma of constipated mice induced by loperamide, including those correlated with the metabolisms of sphingolipid, glycerophospholipid, tryptophan, bile acids, unsaturated fatty acids and amino acids.	[32]
COS	DD: 90% MW: 1500 Da	A preventive and therapeutic effect in mice with DSS-induced chronic UC	Attenuating inflammatory response, ameliorating colonic apoptosis, promoting the proliferation of crypt epithelial cells and modulating gut microbiota	[33]
COS	MW < 1500 Da	Ameliorate metabolic syndrome	Improved their function related to intestinal barrier and glucose transport.	[34]
COS	purity > 95% DD ≥ 90%	Markedly inhibit osteosarcoma cell viability, metastasis, apoptosis and autophagy in vitro and in vivo.	COS-induced autophagy was initiated by the activation of the p53/mTOR pathway.	[35]
COS	NA	Potent immunomodulatory and hepatoprotective effects	COS inhibited the JAK2/STAT1 pathways on M1 macrophages and the JAK1/STAT6 pathways on M2 macrophages in KCs.	[36]
COS	MW: 1100, 2500, 3600 Da DD > 90%	Enhanced antitumor immunity	Inhibited the expression of PD-L1 through the activation of AMPK and the suppression of STAT1 signaling	[37]
COS	MW < 1 kDa purity: 91.0%	Attenuate experimental severe acute pancreatitis	Inhibiting oxidative stress and modulating intestinal homeostasis	[38]

NA: Not available; SCOS: Sulfated Chitooligosaccharide; EVs-COS: Extracellular Vesicles binding with Chitosan Oligosaccharides; COST/COSM: Chitosan Oligosaccharides; COS: Chitosan Oligosaccharides; MW: Weight-average Molecular Weight; DD: Degree of Deacetylation; DP: Degrees of Polymerization.

**Table 3:** Sources and biochemical properties of chitinase.

Organism	Expression Host	Molecular Mass (kDa)	Optimal Temperature (°C)	Optimal pH	Activity (U/mg)	Inhibitor	Activator	Refs.
*Streptomyces albolongus* ATCC 27414	*Escherichia coli* BL21	47	55	5	66.2	Fe^3+^, Cu^2+^, Na^+^, EDTA, SDS	Mn^2+^, Ba^2+^, Na^+^	[57]
*Flavobacterium johnsoniae* UW101	*Escherichia coli Rosetta-gami 2* (DE3)	35.5	40	6	26.2	Ca^2+^, WRK, urea, Hg^2+^	Cu^2+^	[58]
*Trichoderma virens*	*yeast Pichia pastoris*	42	37	4.5	NA	NA	NA	[59]
*Bacillus licheniformis* B307	NA	42	60	6	14.2 U/mL	NA	NA	[60]
*Myxococcus fulvus* screened from soil	*E. coli* DH5a	26.99	35	8	NA	NA	NA	[61]
Marine bacteria DW2	*Antarctic Escherichia coli*	39.5	30	5	7.3	Cr^3+^, Ni^2+^, Fe^3+^, Mn^2+^, Cu^2+^, EDTA, SDS, Hg^2+^, Ag^+^	Ca^2+^, Zn^2+^, Mg^2+^, β-mercaptoethanol	[62]
soil of a mangrove tidal flat	*E. coli* BL21 (DE3)	43	45	NA	0.63	SDS, EDTA, Fe^3+^, Cu^2+^, Mn^2+^, Co^2+^, Ag^+^, Hg^2+^	K^+^, Na^+^	[63]
actinobacterium *Streptomyces olivaceus* (MSU3)	NA	52	40	8	680.0 IU	Hg^2+^, Pb^2+^	Mn^2+^, Cu^2+^, Mg^2+^	[64]
C.shinanensis	*E. coli* BL21DE3-pLysS	58.87	50	7	NA	NA	NA	[65]
*Lysobacter* sp. MK9-1	*Escherichia coli Rosetta-gami B* (DE3)	NA	55	4.5	12	NA	NA	[66]
*Fenneropenaeus merguiensis*	*Escherichia coli*	52	40	6	NA	NA	NA	[67]
*Thermomyces lanuginosus*	NA	18	50	6.5	NA	Cu^2+^, Hg^2+^, EDTA	β-ME	[68]
Chitinolyticbacter meiyuanensis SYBC-H1	*Escherichia coli* BL21	110	50	6	4.1	Cu^2+^, Ni^2+^, Fe^3+^	Fe^2+^, Mg^2+^, Ba^2+,^ Na^+^	[69]
*Acinetobacter indicus* CCS-12	3ZYB medium	50	60	7	480.2	NA	Ca^2+^, Mn^2+^, Mg^2+^, Na^+^, Fe^2+^, Cu^2+^, EDTA and β-mercaptoethanol	[70]
*Fenneropenaeus merguiensis*	NA	52	40	6	NA	NA	NA	[67]

NA: Not available; EDTA: Ethylenediaminetetraaceticacid; SDS: Sodium Dodecyl Sulfate; WRK: Woodward’s reagent K.

**Table 4:** Sources and biochemical properties of CDA.

Organism	Expression Host	Molecular Mass (kDa)	Optimal Temperature (°C)	Optimal pH	Activity (U/mg)	Inhibitor	Activator	Refs.
*Penicillium oxalicum* SAE(M)-51	NA	53	50	9	NA	NA	Cu^2+^, Co^2+^	[92]
*Rhizopus circinans*	NA	75	37	6	NA	Cu^2+^	Mn^2+^, Mg^2+^	[95]
*Aspergillus nidulans*	*Escherichia coli* BL21	24.2	50	8	4.17	NA	NA	[96]
*Arctic deep-sea sediments*	*Escherichia coli* BL21 (DE3)	43	28	7.4	NA	NA	NA	[97]
*Micromonospora aurantiaca*	NA	NA	40	7	NA	Mg^2+^, Cu^2+^, Zn^2+^	Ca^2+^, K^+^	[98]
*Saccharomyces cerevisiae*	NA	NA	50	8	NA	NA	NA	[99]
*marine strain Nitratireductor aquimarinus* MCDA3-3	NA	30	30	8	50	Co^2+^, Ba ^2+^, EDTA	Sr^2+^, Mg^2+^, Na^+^	[100]
*mushroom Coprinopsis cinerea*	NA	27	50	9	693.92 ± 0.30	EDTA, Cu^2+^, Zn^2+^, Al^3+^, Fe^2+^, Ca^2+^	Co^2+^, Mg^2+^	[101]
*Colletotrichum gloeosporioides*	NA	35 kDa and 170 kDa	28	6	0.018	NA	NA	[102]
*Absidia corymbifera* DY-9	NA	NA	55	6.5	NA	acetate, EDTA	Co^2+^, Ca^2+^, Mg^2+^	[103]
*Aspergillus flavus*	NA	28	50	8	NA	NA	Mn^2+^, Zn^2+^	[104]
*Microbacterium esteraromaticum* MCDA02	NA	26	30	8	137.54	Co^2+^, Cd^2+^, EDTA	K^+^,Sr^+^	[105]

NA: Not available; EDTA: Ethylenediaminetetraaceticacid.

**Table 5:** Sources and biochemical properties of chitosanases.

Organism	Expression Host	Molecular Mass (kDa)	Optimal Temperature (°C)	Optimal pH	Activity (U/mg)	Inhibitor	Activator	Refs.
*Gongronella butleri* NBRC105989	NA	47	45	4	NA	NA	NA	[125]
*Staphylococcus capitis*	*Escherichia coli* M15	35	30	7	89.2	EDTA, Ba^2+^, Mg^2+^, Ca^2+^, Ni^2+^, Co^2+^	Mn^2+^, Zn^2+^, Cu^2+^	[126]
blue crab viscera	NA	NA	60	4	100 U/g	Hg^2+^, Cu^2+^	Al^2+^, Ba^2+^, Ca^2+^, K^+^, Mg^2+^, Na^+^, Zn^2+^, Mn^2+^	[127]
*Streptomyces albolongus*	*E. coli* BL21 (DE3)	29.6	50	8		Mg^2+^, Fe^3+^, Zn^2+^, SDS	Mn^2+^, Cu^2+^, Ba^2+^	[128]
*Aspergillus* sp. W-2(CGMCC7018)	*Pichia pastoris* X-33	28	55	6	34	Fe^2+^, Zn^2+^, Ge^2+^, Ni^2+^, Cu^2+^	Ca^2+^, Mn^2+^, Mg^2+^	[122]
Chromobacterium violaceum	*Escherichia coli*	38	50	6.0, 11	10,000	Pb^2+^, Fe^3+^, Hg^2+^, Ni^2+^, Ag^+^, Rb^+^, Fe^2+^, SDS	Ca^2+^, Co^2+^, Cu^2+^, Sr^2+^, Mn^2+^	[67]
*Bacillus amlyoliquefaciens*	*Pichia pastoris*	29	55	6.5	2380.5	NA	NA	[129]
pabuli	*E. coli*	56	45	6	NA	NA	NA	[130]
*Bacillus amyloliquefaciens*	*E. coli* BL21(DE3)-pLys	29	40	5.6	NA	NA	NA	[131]
*deep-sea bacterium Serratia* sp. QD07	*Escherichia coli* BL21(DE3)	27.1	60	5.8	412.6	Cu^2+^, Ni^2+^, Co^2+^	Mg^2+^, Fe^3+^, Ba^2+^, Zn^2+^, EDTA, Fe^2+^, SDS, NH^4+^, Al^3+^, Ca^2+^	[132]
*Aquabacterium* sp. A7-Y	*Escherichia coli* BL21 (DE3)	50.7	40	5	18	Ca^2+^, Mg^2+^, Ni^2+^	Cu^2+^, Mn^2+^	[133]
*Paenibacillus barengoltzii barengoltzii*	*Bacillus subtilis*	NA	70	5.5	360	NA	NA	[134]
*Streptomyces niveus*	*E. coli* BL21(DE3)	29.8	50	6	NA	Fe^3+^	Cu^2+^	[135]
*Penicillium oxalicum* M2	NA	42	60	5.5	60.45	NA	Ca^2+^, Mn^2+^, Tween 20/40/60/80 and Trition X-100, DTT and β-ME)	[136]

NA: Not available; EDTA: Ethylenediaminetetraaceticacid; SDS: Sodium Dodecyl Sulfate.
